# Concerns Regarding the Glorification of Mental Illness on Social Media

**DOI:** 10.7759/cureus.56631

**Published:** 2024-03-21

**Authors:** Jai Ahuja, Palak A Fichadia

**Affiliations:** 1 Psychiatry, Dr. D. Y. Patil Medical College, Hospital & Research Centre, Pune, IND; 2 Medicine, Smt. Nathiba Hargovandas Lakhmichand Municipal Medical College, Ahmedabad, IND

**Keywords:** awareness building, watching movies, mental health professional, social media, mental health illness

## Abstract

In today's world, it is becoming increasingly important to address not only the need for awareness of mental illnesses but also the undue glorification of the same by certain segments of society. Social media has been a major influence on Generation Z in terms of self-diagnosing mental illnesses and romanticizing them. More and more individuals have started recognizing online trends of self-diagnosis of mental illness, and it is increasingly plausible in the process of normalizing the conversation around mental health through memes, TikTok videos, and viral tweets; this has had the paradoxical effect of romanticization of mental illness in certain segments of society.

Through this editorial, we would like to highlight this important issue and urge the mental health community to be cognizant of this phenomenon, lest it curtail society's recent advancements in mental health destigmatisation.

## Editorial

This article will bring attention to a concerning trend observed online, especially among Generation Z (born between 1997-2012), regarding the portrayal and perception of mental illness on social media platforms. As Generation Z health professionals ourselves, we, the authors, believe it is imperative to address the inadvertent glorification of mental health struggles within this demographic.

The advent of social media has undeniably revolutionized the way individuals interact and express themselves. It has fostered a sense of community and bridged societal gaps to an extent that seemed unfathomable only a few decades ago. The mental health challenges propagated by social media have been studied in great detail, with research significantly tying them to heightened levels of anxiety and depression in individuals [1]. This editorial does not aim to talk about the consequences of social media use, which have been extensively studied in the scientific literature; instead, it serves the purpose of a new phenomenon - the glorification of mental illness and viewing it as almost akin to a status symbol by certain segments of Generation Z society [2]. While traditional media has often been criticized for villainizing characters with mental illness, the new-age social media depiction of mental illness carries with it a sense of 'creative mystique' - painting mental illness with a glorified aesthetic in segments of society exposed to such media [2]. According to psychoanalytic theory, an individual's identity is formed by many distinct personalities, implying that identity is numerous rather than unitary. Social media capitalize on this notion, where people create several personalities, some of which are mentally unstable [2]. In addition, on social media sites like Tumblr, the romanticization of mental illness may be especially dangerous because the platform tends to encourage the development of solitary 'echo chambers'. 'Echo chambers' are online communities where people can feed off each other's negativity and reinforce ideas and beliefs through repeated exposure within a closed system. This helps to perpetuate the negative effects of mental illnesses themselves, as well as the romanticization of mental illness [2]. It is worth noting that media surveys have observed that Generation Z is particularly prone to self-diagnosing mental health issues, with 30% having done so in one such survey [3]. Out of these, anxiety (48%) and depression (37%) were the most common. The same survey revealed generational differences in the preferred platform for self-diagnosis, with Generation Z favoring TikTok, in contrast to older generations showing a preference for Facebook.

Through an amalgamation of curated content, relatable memes, and 'literally me' characters, Generation Z finds itself immersed in a digital landscape where mental health challenges are romanticized rather than understood. 

'Literally me' characters are often characters suffering from serious mental illness that have developed a cult following among the masses over the years, propelled by their popularity in internet meme culture. An example would be Patrick Bateman from the psychological thriller" American Psycho," or the titular character from the film 'Joker' who, despite significant mental illness, is often glamourized and envied in modern society [4]. Closer home in India, 'Kabir Singh', a character with schizotypal personality disorder and substance use disorder who shares his name with the film he is a part of, has propagated the overcompensation of masculine identities in Indian society and has been found to affect attitudes in heterosexual relationships significantly [5].

Recent studies, including the BBC experiment highlighting the human tendency to manufacture problems, shed light on the psychological underpinnings driving the phenomenon of mental illness glorification [6]. The allure of negativity, packaged in aesthetically pleasing edits and shared experiences, captivates young audiences, blurring the lines between universal emotions and genuine mental health symptoms. Consequently, impressionable individuals may self-diagnose or adopt personality traits associated with fictional characters, leading to potential misinterpretations and worsening of existing challenges [7]. A self-diagnosis of mental illnesses has been frequently self-reported by members of the involuntary celibate community who have also been extensively linked to mass shooter violence [8]. According to one study, 95% and 93% of this community had self-diagnosed themselves with depression and anxiety, respectively [8].

One can speculate that in the process of normalization and destigmatization, the unintended consequence of using mental illness as a get-out-of-jail-free card, i.e., an exoneration mechanism, has emerged.

Health professionals must navigate this complex terrain with empathy and discernment. While the destigmatization of mental illness remains a crucial objective, we must exercise caution to ensure that the conversation around mental health does not inadvertently veer into the realm of romanticization. We advocate for a balanced approach that acknowledges moments of well-being while remaining sensitive to the realities of those grappling with genuine challenges.

In other words, as much as it is okay to not be okay, it is also okay to be okay.
